# Peculiarities in the Therapeutic Action of Nux Vomica in Different Individuals

**Published:** 1888-03

**Authors:** Chas. Chassaignac

**Affiliations:** New Orleans


### PECULIARITIES IN THE THERAPEUTIC ACT ON OF NUX VOMICA IN DIFFERENT INDIVIDUALS.

BY CHAS. CHASSAIGNAC, M. D., NEW ORLEANS.

We wish to call the attention of the society to night to the wide range of intensity with which nux vomica and strychnia act upon different individuals.

It will be remembered that some few meetings back the discussion turned upon the effect of ordinary doses of strychnia, and the conclusion was reached that the alkaloid in question was usually employed in doses too small for any effect to be obtained. Large doses were advocated, and several instances were mentioned, in support of this opinion, in which enormous doses were well borne.

The cases we report to-night show the other side of the matter and are to be added to the long list of analogous ones already contained in the literature of therapeutics and materia medica.

CASE I.—A lady about forty years old, of generally good health, consulted the writer for what was determined to be a mild form of malaria, without marked paroxysms, but producing some nervous depression. A tonic and antiperiodic was prescribed. each dose of which included one-quarter of a grain of nux vomica. An hour after taking the second dose she suddenly had what she feared to be an attack of incipient paralysis, and we were hurriedly summoned. She was found in bed, much agitated, complaining of lightness of head, and explained that, while walking across the room she suddenly found that she had not full control of her lower limbs, which "jerked" as she attempted to move and caused her to fall. She was unable to rise without assistance, and had to be led to her bed. We reassured her, prescribed full doses of bromide and in a few hours she had entirely recovered. Needless to add that the first medicine was discontinued.

CASE 2—Was that of a young married woman who was pulled down by fatigue and grief. We gave her a sample one ounce bottle of the hematic syrup prepared by Parke, Davis & Co., containing one-sixteenth of a grain of strychnia to the ounce. The second day after we were called in haste to find her in what appeared to be a very serious condition. She was delirious, talking incessantly, complaining of lightness and emptiness of the head ; touching the latter she would exclaim, "It's empty ; I tell you it's empty !" She showed a degree of opisthotonos, had severe contractions of muscles of the neck, arms and legs, which caused intense suffering. During intervals between contractions there was great tremulousness and she complained of numbness of extremities. Pulse was small and frequent Respiration hurried and jerky. There could be no mistake in the diagnosis, and we looked for the vial of hematic syrup to see if an excess had been taken, but found that less than half of it was missing, so that these symptoms were produced by less than one-thirty-second of a grain of strychnia, taken in divided doses. Bromide of potassium and chloral hydrate were administered in good doses every hour. It was only after the scond dose that the symptoms began to abate, and only after six or seven hours that she commenced to sleep and was relieved of the severe symptoms. Next day she still had slight muscular twichings and some numbness and soreness of the extremities. The bottle of tonic was made to disappear, and this patient was put down as one to whom strychnia will never again be administered as long as she is under our care.

These two cases are sufficient to show us how careful we must be in prescribing strychnia. One patient was very much alarmed and had distressing symptoms after the administration of a half grain of extract of nux vomica in two doses ; the other was made seriously sick and suffered greatly from taking less than a thirty-second of a grain of strychnia in thirty-six hours. Imagination had nothing to do with the symptoms, for in neither case did the patient suspect what she was taking.

When taken in connection with the cases referred to as having been reported at a previous meeting, these illustrate how extremely strychinia can vary in its effect upon different individuals. —*New Orleans Medical Journal.*

